# Perceived public transport infrastructure modifies the association between public transport use and mental health: Multilevel analyses from the United Kingdom

**DOI:** 10.1371/journal.pone.0180081

**Published:** 2017-08-16

**Authors:** Xiaoqi Feng, Zhiqiang Feng, Thomas Astell-Burt

**Affiliations:** 1 Population Wellbeing and Environment Research Lab (PowerLab), School of Health and Society, Faculty of Social Sciences, University of Wollongong, Wollongong, Australia; 2 Menzies Centre for Health Policy, School of Public Health, University of Sydney, Sydney, Australia; 3 School of Geosciences, Drummond Library, Surgeon’s Square, Drummond Street, Edinburgh, Scotland, United Kingdom; Iranian Institute for Health Sciences Research, ISLAMIC REPUBLIC OF IRAN

## Abstract

**Aims:**

Investments to promote public transport utilisation are being championed to achieve sustainable development, but the potential co-benefits for mental health are comparatively under-researched. We hypothesised that frequent users of public transport would be more likely to have better mental health (possibly due to increased levels of physical activity), but among the more frequent users, less favourable perceptions of public transport infrastructure (PPTI) could have a negative influence on mental health.

**Methods:**

Multilevel linear and logistic regressions were fitted on 30,214 participants in the UK Household Longitudinal Study with lagged PPTI and confounder measures at baseline and indicators of active travel and mental health (General Health Questionnaire (GHQ), SF-12 Mental Component Scale (MCS) and the Warwick Edinburgh Mental Well Being Scale (WEMWBS)) at follow-up.

**Results:**

Compared to participants expressing poor PPTI, those who felt it was excellent were 1.29 (95%CI 1.15, 1.45) times more likely to be frequent users of public transport and 1.53 (95%CI 1.33, 1.76) times more likely to choose to walk or cycle journeys of less than two to three miles. Frequent use of public transport was found to be consistently associated with better mental health for GHQ caseness (OR 0.85, 95%CI 0.79, 0.91), GHQ score (coefficient -0.28, 95%CI -0.41, -0.16), MCS (coefficient 0.45, 95%CI 0.23, 0.66), and WEMWBS (coefficient 0.30, 95%CI 0.19, 0.40). Among frequent users of public transport, participants expressing poor PPTI were 1.46 (95%CI 1.11, 1.93) times more likely to report poorer mental health according to the GHQ caseness indicator, compared to frequent users that regarded PPTI as excellent. Similar results were observed for the other indicators of mental health.

**Conclusions:**

These findings indicate that while the provision of public transport infrastructure is a necessary pre-condition for stimulating population increases in physical activity, PPTI improvements needs to be prioritised to leverage the full mental health-related co-benefits of active travel.

## Introduction

Despite considerable investment in scientific research worldwide, there remains little evidence of interventions that promote physical activity in the medium-to-long term [1]. Up till relatively recently, this evidence has been largely focussed upon person-level factors and the role of practitioners, but now there is a wider appreciation that many decisions made outside the more traditional purview of the health sector are fundamental determinants of healthy, active and long lives [2]. If the environments that we design and build go on to shape where people live and what they can choose to do with their time [3], however, this suggests radical (i.e. population-level [4]) change is a necessary pre-condition to stimulate positive and sustained behavioural change [5, 6]. It is in this context that the co-benefits of engendering active travel within daily life as a means for long-term improvements in physical activity and associated health status, such as the prevention of cardiometabolic diseases like type 2 diabetes mellitus, come to the fore [7–11].

Active travel generally equates to walking for transport and cycling, but also to the use of public transport, for which physical activity is a clear factor, unlike when travelling in cars. The determination of active travel via public transport is likely to be influenced, therefore, by a complex interplay between person-level characteristics and the availability of public transport infrastructure [12]. Journeys over short distances, for example, may be easily traversable by walking or cycling, but longer journeys usually invoke a choice to go by car or, if sufficient infrastructure is available, via public transport. It is the word ‘sufficient’ that is problematic, however, given that just because public transport infrastructure is available does not mean it is deemed to be of an acceptable standard for use. Perceptions of public transport that are likely to matter for whether a person considers it an option for active travel probably include a lack of information, high cost, if it is infrequent or unreliable, difficult to access, or perceived unsafe [13, 14].

Thus, while active travel via public transport may lead to gains in physical activity *per se* relative to travelling by car, if a person has little choice but to use public transport they perceive as of unacceptable quality, this may in fact be an important source of psychosocial stress that accumulates and repeatedly insults over time, perhaps resulting in loss of a sense of control over one’s life, with negative downstream impacts on psychological and physiological health [15–17]. Research on this issue has been scant, but what evidence there is remains equivocal. A recent study, for example, reported benefits of active travel for physical health but null findings for mental health [18]. Meanwhile, previous research has suggested that the psychological implications of using public transport as opposed to cars may potentially include reduced feelings of autonomy, mastery, prestige and self-esteem [19]. These findings demonstrate that the relation between active travel and mental health is not straightforward, yet has clear health and policy relevance. Accordingly, the purpose of our study was to examine the interplay between perceptions of public transport infrastructure (PPTI, hereafter) and use of public transport for a range of mental health indicators.

## Method

### Setting

The study is set in the United Kingdom of Great Britain and Northern Island. The UK is an ideal setting for this research with just over half of all distances travelled as car drivers and about 27% in addition as car passengers in 2010 [20]. Although there has been growth in use of trains, this has reportedly come from more people initiating travel by public transport rather than greater frequency of use among existing rail users [20]. This growth in demand has manifested after (and perhaps in spite of) the infamous privatisation of the UK rail network [21, 22]. Importantly, there are significant geographic variations in public transport availability, with rail travel more common car travel less common among residents in London compared to those outside the capital [20].

### Design

PPTI was conceptualised as an antecedent of public transport use on the proviso that those who judge public transport to be of poor quality are likely to select alternative modes of transportation. PPTI was not considered to be a direct antecedent of mental health, however, it was considered to be an effect modifier of the potentially causal effect of public transport use on mental health. For the many people who do use public transport, this experience is hypothesised to be a variable source of psychosocial stress depending upon the level of PPTI. Public transport use, PPTI and mental health are all confounded by various indicators as illustrated.

### Data

The data used for this study was an extract from the UK Household Longitudinal Study (UKHLS), for which information on the design, sampling and content are already available [23, 24]. In brief, the UKHLS is a large panel study consisting of waves collected over two years or 24 months, with the first wave in beginning in 2009–10. Interviews with participants took place annually using computer assisted personal interviewing (CAPI), rendering a pattern of overlapping waves as part of the design. One household member completed the household enumeration grid and the household interview. Each resident of an enumerated household aged 16 years or older was administered an individual adult interview and a self-completed questionnaire. Questionnaire instruments and survey materials were translated into nine languages. The overall response rate at wave 1 was 57.3% (n = 50,199), which is suggested to be typical for multi-purpose surveys of this type in the UK. The data extract used in this study was from waves 3 (2011–2012) and 4 (2012–2013), with response rates of 76.1% and 80.7% respectively,[24] due to the availability of relevant indicators for this study. Herein, data from wave 3 (PPTI and confounders) are referred to as ‘baseline’ whereas data from wave 4 (mental health and public transport use) is labelled ‘follow-up’.

### Mental health

Four contrasting indicators of mental health were examined at follow-up in order to triangulate the potential influence of PPTI and public transport use. The first two indicators were based upon a measure of minor psychiatric morbidity, derived from the 12-item General Health Questionnaire (GHQ) [25]. The GHQ-12 was developed as a screening instrument for use in primary care settings. It includes questions on concentration, sleep loss due to worry, perception of role, capability in decision making, whether constantly under strain, perception of problems in overcoming difficulties, enjoyment of day-to-day activities, coping resources, loss of confidence, self-worth, general happiness and whether suffering depression or unhappiness [26]. Responses to these items are summed to construct a continuous measure. The first mental health indicator was a binary variable denoting participants with GHQ scores of 4 or greater were classified as having clinically significant minor psychiatric morbidity, compared with those scoring less than 4. This threshold has been identified as appropriate within the UK population [27]. The second indicator of mental health was the GHQ-12 score, a normally-distributed (i.e. continuous) variable that is derived from the same questions as the GHQ-12 [25, 26].

The third indicator of mental health used in this study was the mental component scale (MCS) of the 12-item Short Form Health Survey (SF-12), a commonly used measure of general health and functioning in epidemiological research [24]. The SF-12 is an abbreviated form of the 36-item instrument (SF-36) designed to assess general self-rated health, physical and psychological symptoms, and limitations in everyday activity due to physical and mental health over the previous 4 weeks [28]. The MCS was developed using item weights based on an orthogonal factor rotation and scores have been used to successfully discriminate between the presence and severity of mental disorders in clinically defined groups of adults, though there is no widely accepted screening cut-off score for probable diagnosis of any mental disorder [29]. As such, the MCS was used as a continuous outcome variable with normal distribution in this study.

The fourth indicator of mental health used was the Warwick Edinburgh Mental Well Being Scale (WEMWBS) [30]. Whereas the GHQ and SF-12-based MCS are indicators that focus upon negative aspects of mental health, WEMWBS provides a counterpoint by emphasising positive mental health (a term often used interchangeably with ‘mental wellbeing’). WEMWBS attempts to capture affective-emotional aspects, cognitive-evaluative dimensions and psychological functioning by focussing entirely on the positive and, therefore, free of so-called ‘ceiling effects’. WEMWBS has been shown to have high levels of internal consistency and reliability against accepted criteria [31]. In this study, WEMWBS follows a normal distribution and is used as a continuous outcome variable.

### Active travel

Two indicators of active travel were obtained at follow-up. Participants were asked ‘could you tell me how often you personally’: (i) ‘use public transport (e.g. bus, train) rather than travel by car?’; (ii) ‘walk or cycle for short journeys less than 2 or 3 miles?’ We constructed binary variables for each, distinguishing between participants in active travel responding ‘always’, ‘very often’ or ‘quite often’ (referred to hereafter as ‘frequent’), in comparison to those responding ‘not very often’ or ‘never’ (i.e. ‘infrequent’).

### Perceptions of public transport infrastructure (PPTI)

PPTI indicators were only available at baseline. All participants were asked ‘How would you rate public transport services in your local area?’ Participants had the option to rate public transport as ‘excellent’, ‘very good’, ‘fair’, ‘poor’, or ‘no opinion’.

### Confounders

A range of demographics (age, gender), socioeconomic factors (highest educational qualification and economic status e.g. employment, retired), physical functioning, and urban/rural status of the area of residence were taken into account as potential confounders of public transport availability and mental health. These confounders were all measured at baseline, to avoid the potential for reverse causation with public transport use and mental health measured at follow-up.

### Analysis

The data extract was restricted to participants from baseline (n = 54,781) who were successfully followed up (n = 42,115) and did not move home in between (n = 35,931). This sampling strategy was implemented to minimise the likelihood of a change in provision of local public transport, so that the PPTI variable measured at baseline could be expected to be consistent also at follow-up. This sample was further reduced to those participants with full mental health data for each of the four indicators (n = 30,214). Logistic regression was used to examine the patterning of missing mental health outcome data against PPTI, active travel and the aforementioned confounders.

A three-level multilevel model was employed to account for the nesting of persons (level 1, n = 30,214) within households (level 2, n = 19,528) due to the nature of the UKHLS. Regional variation, such as the differences in public transport infrastructure within London compared with other areas of the UK, were also taken into account (level 3, n = 12 Government Office Regions). An average of 1.5 participants were clustered within households (min = 1, max = 7), whereas the equivalent average for regions was 2517.8 (min = 1161, max = 3685). Multilevel logistic regression was used to analyse the patterning of poor PPTI across each of the confounding variables. The same regression technique was then applied to assess the degree of association between PPTI and active travel one year later, adjusting for the confounders. Multilevel logistic and linear regressions were then used to examine the interplay of PPTI and public transport use for predicting each of the four mental health outcomes (logistic regression for GHQ-12 caseness, linear regression for the GHQ-12 score, MCS and WEMWBS). In each of these models, the variance partition coefficient and, where relevant, the median odds ratio were calculated to report variation in each outcome across households and regions [32]. All models were run in MLwIN v.2.31 [33].

## Results

The odds of missing mental health indicator data were lower among women than men (OR = 0.92, 95%CI 0.86, 0.97), higher among older participants (e.g. ≥70y compared with 15-19y OR = 2.78, 95%CI 2.40, 3.22), higher among participants without qualifications (OR = 4.29, 95%CI 5.58, 7.90), higher among the unemployed (OR = 1.81, 95%CI 1.59, 2.07), the retired (OR = 2.14, 95%CI 2.00, 2.28), the long-term sick or disabled (OR = 2.64, 95%CI 2.31, 3.01), those reporting more favourable PPTI (e.g. excellent versus poor OR = 1.21, 95%CI 1.07, 1.37), and participants who often chose to use public transport (OR = 1.37, 95%CI 1.28, 1.47). Missing data was less common among participants with higher physical functioning (e.g. highest versus lowest quintile OR = 0.018, 95%CI 0.016, 0.021), living in rural areas compared with urban (OR = 0.80, 95%CI 0.75, 0.85), and who chose to walk or cycle short journeys less than two to three miles (OR = 0.86, 95%CI 0.81, 0.92).

Descriptive findings concerning the patterning of poorer mental health with respect to sociodemographic characteristics were in line with previous literature [34, 35]. Poorer mental health was observed among women compared with men across all four indicators (Table 1). Mental health was also found to vary in a curvilinear fashion by age, with poorer levels observed among younger to middle-aged adults. Participants with lower levels of education, the unemployed and retired, those with lower physical functioning and those living in urban areas also tended to have poorer mental health. Participants who often chose to walk or cycle for short journeys of less than two to three miles tended to have better mental health than those who did not. In contrast, participants who often chose to use public transport rather than a car tended to have poorer mental health, especially demonstrable with the GHQ caseness variable. These findings are broadly in line with those more generally that suggest higher levels of physical activity are associated with more favourable levels of mental health (e.g. [36]). New findings from this descriptive analysis suggest that participants with better PPTI tended to have better mental health, most clearly observable using the GHQ caseness indicator.

**Table 1:** Descriptive statistics and patterning of mental health variables.

	N	%	GHQ caseness	GHQ score	MCS	WEMWBS
			% ≥ 4 (95%CI)	Mean (95%CI)	Mean (95%CI)	Mean (95%CI)
Gender						
male	13,127	43.4	14.57 (13.98, 15.19)	10.29 (10.20, 10.38)	50.78 (50.61, 50.94)	24.88 (24.80, 24.95)
female	17,090	56.6	20.63 (20.03, 21.25)	11.44 (11.36, 11.53)	48.84 (48.69, 48.98)	24.62 (24.55, 24.68)
Age group						
16-19y	1,702	5.6	19.86 (18.03, 21.82)	10.40 (10.15, 10.66)	48.98 (48.53, 49.43)	24.32 (24.11, 24.53)
20-24y	1,406	4.7	20.84 (18.80, 23.04)	11.11 (10.83, 11.39)	48.14 (47.64, 48.63)	24.02 (23.78, 24.25)
25-29y	1,735	5.7	20.29 (18.46, 22.25)	11.16 (10.91, 11.41)	47.56 (47.11, 48.00)	24.02 (23.81, 24.23)
30-34y	2,348	7.8	19.63 (18.08, 21.29)	11.12 (10.90, 11.33)	47.98 (47.59, 48.36)	24.38 (24.20, 24.56)
35-39y	2,655	8.8	19.17 (17.72, 20.71)	11.19 (10.99, 11.39)	48.27 (47.90, 48.63)	24.43 (24.26, 24.60)
40-44y	3,166	10.5	19.49 (18.15, 20.91)	11.36 (11.17, 11.55)	48.43 (48.10, 48.76)	24.41 (24.26, 24.56)
45-49y	3,036	10.1	19.76 (18.38, 21.22)	11.52 (11.33, 11.71)	49.09 (48.75, 49.42)	24.35 (24.19, 24.51)
50-54y	2,896	9.6	21.72 (20.26, 23.26)	11.68 (11.48, 11.87)	48.98 (48.63, 49.32)	24.32 (24.16, 24.48)
55-59y	2,545	8.4	19.17 (17.69, 20.75)	11.32 (11.11, 11.53)	49.84 (49.47, 50.21)	24.75 (24.58, 24.92)
60-64y	2,637	8.7	14.52 (13.23, 15.92)	10.42 (10.21, 10.62)	51.54 (51.17, 51.90)	25.49 (25.32, 25.65)
65-69y	2,345	7.8	12.37 (11.09, 13.76)	10.01 (9.80, 10.23)	52.70 (52.32, 53.09)	25.90 (25.72, 26.08)
70y or older	3,746	12.4	12.79 (11.76, 13.90)	10.05 (9.88, 10.22)	52.40 (52.10, 52.71)	25.56 (25.42, 25.70)
Highest educational qualifications						
Degree	7,143	23.6	16.39 (15.55, 17.27)	10.61 (10.48, 10.73)	49.91 (49.69, 50.14)	25.41 (25.31, 25.52)
Other higher degree	3,590	11.9	16.94 (15.74, 18.20)	10.83 (10.65, 11.01)	50.08 (49.76, 50.39)	25.04 (24.89, 25.18)
A-level	6,094	20.2	17.77 (16.83, 18.75)	10.86 (10.73, 11.00)	49.58 (49.34, 49.82)	24.69 (24.57, 24.80)
GCSE	6,495	21.5	18.89 (17.96, 19.86)	11.09 (10.96, 11.22)	49.36 (49.12, 49.59)	24.26 (24.15, 24.37)
Other qualification	2,900	9.6	18.79 (17.41, 20.26)	11.15 (10.95, 11.34)	50.13 (49.78, 50.48)	24.36 (24.20, 24.52)
No qualification	3,624	12.0	20.28 (19.00, 21.62)	11.45 (11.27, 11.62)	49.30 (48.99, 49.62)	24.33 (24.18, 24.47)
Missing	371	1.2	18.87 (15.20, 23.17)	10.75 (10.20, 11.29)	48.81 (47.83, 49.79)	24.32 (23.87, 24.77)
Economic status						
employed	17,377	57.5	16.03 (15.49, 16.59)	10.63 (10.56, 10.71)	49.93 (49.80, 50.07)	24.87 (24.81, 24.94)
unemployed	1,306	4.3	30.09 (27.66, 32.64)	12.90 (12.62, 13.18)	46.02 (45.52, 46.52)	22.66 (22.43, 22.90)
retired	7,496	24.8	13.78 (13.02, 14.58)	10.23 (10.11, 10.34)	52.07 (51.86, 52.28)	25.61 (25.51, 25.70)
family care	1,593	5.3	25.30 (23.22, 27.49)	12.31 (12.05, 12.56)	46.70 (46.25, 47.16)	23.78 (23.57, 23.99)
student	1,363	4.5	19.96 (17.92, 22.16)	10.70 (10.42, 10.98)	48.52 (48.02, 49.01)	24.52 (24.29, 24.75)
sick/disabled	956	3.2	54.39 (51.22, 57.53)	17.41 (17.07, 17.74)	38.17 (37.58, 38.76)	20.09 (19.82, 20.37)
don't know	126	0.4	25.40 (18.56, 33.71)	12.09 (11.18, 13.00)	47.50 (45.88, 49.11)	23.84 (23.08, 24.60)
Physical functioning (quintiles)						
1 (low)	2,686	8.9	38.38 (36.56, 40.24)	14.45 (14.25, 14.64)	47.29 (46.94, 47.63)	22.92 (22.76, 23.09)
2	6,755	22.4	21.20 (20.24, 22.19)	11.64 (11.52, 11.76)	50.37 (50.16, 50.59)	24.17 (24.07, 24.28)
3	7,018	23.2	13.78 (12.99, 14.61)	10.24 (10.12, 10.36)	50.82 (50.61, 51.04)	24.87 (24.77, 24.97)
4	7,648	25.3	7.37 (6.81, 7.98)	8.94 (8.82, 9.05)	52.89 (52.69, 53.10)	25.97 (25.87, 26.07)
5 (high)	6,110	20.2	23.65 (22.60, 24.73)	11.96 (11.83, 12.08)	44.63 (44.40, 44.86)	24.42 (24.31, 24.53)
Urban/rural						
urban	22,156	73.3	18.85 (18.34, 19.37)	11.07 (11.00, 11.14)	49.29 (49.16, 49.42)	24.61 (24.55, 24.67)
rural	8,058	26.7	15.66 (14.88, 16.47)	10.60 (10.49, 10.72)	50.75 (50.54, 50.96)	25.05 (24.95, 25.15)
missing	3	0.0	33.33 (4.34, 84.65)	11.67 (5.57, 17.76)	53.56 (42.70, 64.42)	28.00 (22.96, 33.04)
Perceptions of public transport infrastructure						
Poor	3,606	11.9	21.33 (20.02, 22.69)	11.50 (11.32, 11.67)	49.22 (48.91, 49.54)	24.69 (24.54, 24.83)
Fair	7,552	25.0	18.33 (17.47, 19.22)	11.07 (10.95, 11.19)	49.33 (49.11, 49.55)	24.44 (24.34, 24.54)
Very good	12,334	40.8	16.98 (16.33, 17.65)	10.76 (10.66, 10.85)	49.90 (49.73, 50.07)	24.80 (24.72, 24.88)
Excellent	3,384	11.2	18.17 (16.91, 19.51)	10.67 (10.49, 10.85)	49.81 (49.48, 50.13)	25.12 (24.97, 25.27)
No opinion	3,015	10.0	17.08 (15.78, 18.47)	11.00 (10.81, 11.19)	50.06 (49.72, 50.41)	24.79 (24.63, 24.95)
Don't know/missing/proxy	326	1.1	19.02 (15.12, 23.65)	11.10 (10.52, 11.69)	49.53 (48.49, 50.57)	24.67 (24.19, 25.16)
Choose to walk or cycle for short journeys less than 2 or 3 miles						
not often or never	18,258	60.4	18.01 (17.46, 18.58)	10.99 (10.91, 11.07)	49.76 (49.62, 49.90)	24.68 (24.62, 24.75)
very often or more	10,567	35.0	16.17 (15.48, 16.89)	10.57 (10.47, 10.67)	49.91 (49.73, 50.09)	24.99 (24.90, 25.07)
missing	1,392	4.6	31.68 (29.29, 34.17)	13.19 (12.91, 13.47)	46.91 (46.40, 47.41)	23.36 (23.13, 23.59)
Choose to use public transport rather than car						
not often or never	23,186	76.7	16.93 (16.46, 17.42)	10.82 (10.75, 10.89)	50.00 (49.87, 50.12)	24.83 (24.77, 24.88)
very often or more	5,380	17.8	20.43 (19.37, 21.53)	11.10 (10.96, 11.25)	48.93 (48.67, 49.19)	24.49 (24.38, 24.61)
missing	1,651	5.5	25.08 (23.04, 27.22)	12.18 (11.92, 12.44)	47.65 (47.18, 48.11)	24.12 (23.91, 24.34)

GHQ = General Health Questionnaire | MCS = SF-12 Mental Health Component | WEMWBS = Warwick Edinburgh Mental Well Being Scale

The odds of reporting poor PPTI were significantly higher among women than men, but did not appear to vary significantly by age or economic status (Table 2). Compared to participants with degrees, those with non-university educational qualifications were less likely to report poor PPTI. Participants with higher levels of physical functioning were significantly less likely to report poor PPTI, whereas those in rural areas with over four times more likely to report poor PPTI than their urban counterparts. PPTI was found to vary both regionally (VPC = 1.3%) and, especially, at the household level (VPC = 27.4%).

**Table 2:** Patterning of poor perceptions of public transport infrastructure.

Fixed Part	OR (95%CI)
Gender (ref: male)	
female	1.10 (1.02, 1.19)
Age group (16-19y)	
20-24y	1.21 (0.92, 1.58)
25-29y	0.84 (0.63, 1.12)
30-34y	0.73 (0.55, 0.97)
35-39y	0.82 (0.62, 1.07)
40-44y	0.83 (0.64, 1.08)
45-49y	1.03 (0.79, 1.34)
50-54y	1.03 (0.79, 1.33)
55-59y	1.26 (0.97, 1.64)
60-64y	1.03 (0.78, 1.37)
65-69y	0.92 (0.68, 1.26)
70y or older	0.86 (0.63, 1.17)
Highest educational qualification (ref: Degree)	
Other higher degree	0.92 (0.81, 1.06)
A-level	0.85 (0.76, 0.96)
GCSE	0.81 (0.72, 0.91)
Other qualification	0.78 (0.67, 0.91)
No qualification	0.73 (0.63, 0.85)
Missing	1.00 (0.70, 1.43)
Economic status (ref: employed)	
unemployed	1.14 (0.93, 1.39)
retired	0.84 (0.71, 0.99)
family care	0.91 (0.75, 1.10)
student	0.86 (0.66, 1.12)
sick/disabled	0.89 (0.70, 1.13)
don't know	1.13 (0.64, 2.00)
Physical functioning (ref: 1 (low))	
2	0.79 (0.68, 0.93)
3	0.72 (0.61, 0.85)
4	0.63 (0.54, 0.75)
5 (high)	0.73 (0.61, 0.86)
Urban/rural (ref: urban)	
rural	4.49 (4.10, 4.90)
**Random Part**	
Regional variance (standard error)	0.061 (0.027)
VPC, MOR	1.3%, 1.27
Household variance (standard error)	1.267 (0.074)
VPC, MOR	27.4%, 2.93

OR = Odds Ratio | 95%CI = 95% Confidence Interval

VPC = Variance Partition Coefficient | MOR = Median Odds Ratio

**Fig 1:**
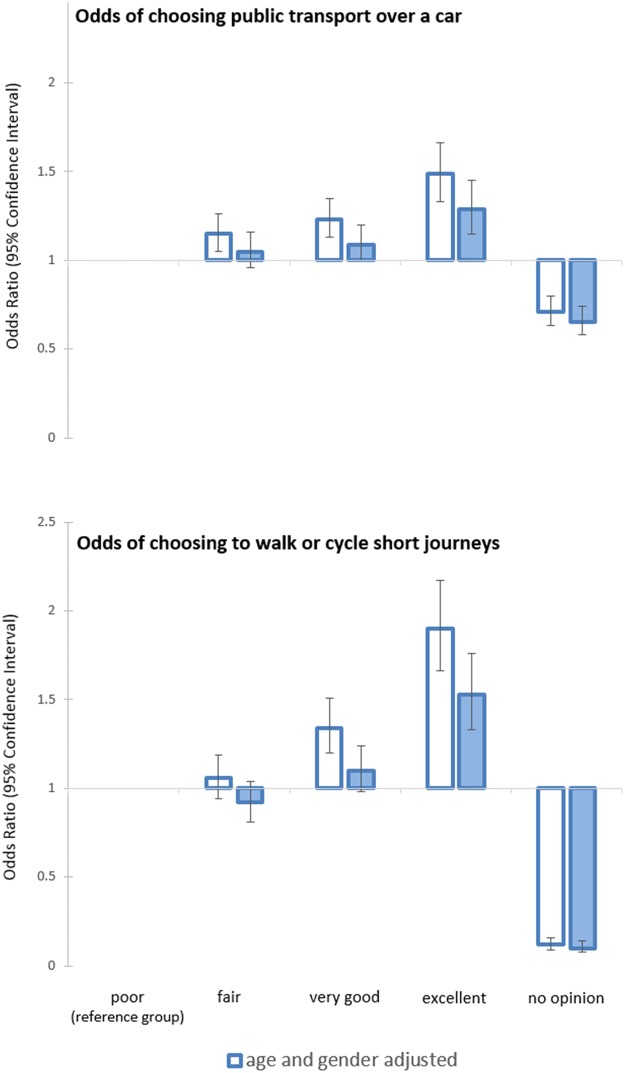
Association between public transport use and perceptions of public transport infrastructure.

The age and gender adjusted odds of choosing to use public transport over a car often, and also to walk or cycle short journeys of less than two to three miles were higher among participants reporting more favourable PPTI (Table 3). This association was partially attenuated, but remained notable after adjusting for socioeconomic confounders, physical functioning and urban/rural variable. These results can also be seen in Fig 1. Contrasts were observed in the variances of each outcome variable. Whereas 1.3% of variation in frequent use of public transport manifested regionally and 9.1% at the household scale, the equivalent for choosing to walk or cycle short journeys was 8.7% between regions and 11.9% between households. These regional variations were only partially explained by adjustment for socioeconomic factors, physical functioning and urban/rural, with rural dwellers less likely to be active travellers for both outcomes.

**Table 3:** Association between public transport use and perceptions of public transport infrastructure.

	Frequent user of public transport		Frequently chooses to walk or cycle short journeys	
	Model 1	Model 2	Model 1	Model 2
**Fixed Part**	Odds Ratio (95% Confidence Interval)			
Gender (ref: male)				
female	0.86 (0.82, 0.91)	0.86 (0.81, 0.90)	1.19 (1.12, 1.27)	1.18 (1.11, 1.27)
Age group (16-19y)				
20-24y	0.70 (0.60, 0.81)	0.80 (0.67, 0.96)	0.67 (0.56, 0.79)	0.94 (0.77, 1.14)
25-29y	0.68 (0.59, 0.79)	0.83 (0.70, 0.99)	0.45 (0.38, 0.54)	0.71 (0.58, 0.87)
30-34y	0.66 (0.57, 0.75)	0.83 (0.70, 0.98)	0.29 (0.24, 0.34)	0.46 (0.37, 0.57)
35-39y	0.62 (0.54, 0.71)	0.81 (0.68, 0.96)	0.31 (0.27, 0.37)	0.51 (0.42, 0.62)
40-44y	0.53 (0.46, 0.60)	0.69 (0.59, 0.82)	0.26 (0.22, 0.30)	0.41 (0.33, 0.50)
45-49y	0.52 (0.46, 0.59)	0.71 (0.60, 0.84)	0.25 (0.21, 0.29)	0.40 (0.33, 0.49)
50-54y	0.54 (0.48, 0.62)	0.75 (0.63, 0.89)	0.30 (0.26, 0.35)	0.46 (0.38, 0.57)
55-59y	0.61 (0.53, 0.70)	0.84 (0.71, 1.00)	0.30 (0.26, 0.36)	0.45 (0.36, 0.55)
60-64y	0.56 (0.49, 0.64)	0.74 (0.61, 0.89)	0.39 (0.34, 0.46)	0.54 (0.43, 0.67)
65-69y	0.55 (0.48, 0.63)	0.69 (0.57, 0.85)	0.43 (0.37, 0.50)	0.56 (0.44, 0.72)
70y or older	0.43 (0.38, 0.49)	0.57 (0.46, 0.70)	0.55 (0.48, 0.63)	0.68 (0.53, 0.87)
Perceptions of public transport (ref: poor)				
fair	1.15 (1.05, 1.26)	1.05 (0.96, 1.16)	1.06 (0.94, 1.19)	0.92 (0.81, 1.04)
very good	1.23 (1.13, 1.35)	1.09 (1.00, 1.20)	1.34 (1.20, 1.51)	1.10 (0.98, 1.24)
excellent	1.49 (1.33, 1.66)	1.29 (1.15, 1.45)	1.90 (1.66, 2.17)	1.53 (1.33, 1.76)
no opinion	0.71 (0.63, 0.80)	0.65 (0.58, 0.74)	0.12 (0.09, 0.16)	0.10 (0.08, 0.14)
missing	0.56 (0.42, 0.76)	0.51 (0.38, 0.69)	0.07 (0.03, 0.21)	0.06 (0.02, 0.19)
Highest educational qualification (ref: Degree)				
Other higher degree		0.96 (0.87, 1.05)		0.86 (0.76, 0.98)
A-level		0.95 (0.88, 1.03)		0.87 (0.78, 0.97)
GCSE		1.00 (0.92, 1.08)		1.00 (0.90, 1.11)
Other qualification		1.03 (0.93, 1.14)		1.20 (1.05, 1.37)
No qualification		1.05 (0.95, 1.17)		1.51 (1.34, 1.72)
Missing		1.08 (0.84, 1.38)		1.20 (0.91, 1.60)
Economic status (ref: employed)				
unemployed		1.99 (1.75, 2.26)		2.18 (1.88, 2.52)
retired		1.28 (1.14, 1.45)		1.27 (1.09, 1.49)
family care		1.44 (1.28, 1.62)		1.00 (0.85, 1.17)
student		1.48 (1.26, 1.75)		2.23 (1.86, 2.66)
sick/disabled		1.01 (0.83, 1.23)		1.86 (1.50, 2.30)
don't know		1.18 (0.79, 1.75)		0.84 (0.47, 1.48)
Physical functioning (ref: 1 (low))				
2		1.90 (1.66, 2.17)		1.06 (0.92, 1.22)
3		2.30 (2.01, 2.64)		1.08 (0.93, 1.25)
4		2.56 (2.23, 2.93)		0.96 (0.83, 1.12)
5 (high)		2.64 (2.30, 3.04)		0.96 (0.82, 1.13)
Urban/rural (ref: urban)				
rural		0.73 (0.68, 0.78)		0.50 (0.45, 0.55)
**Random Part**				
Regional variance (standard error)	0.048 (0.020)	0.036 (0.016)	0.359 (0.148)	0.294 (0.122)
VPC, MOR	1.3%, 1.23	1.0%, 1.20	8.7%, 1.77	7.2%, 1.68
Household variance (standard error)	0.333 (0.032)	0.329 (0.033)	0.491 (0.050)	0.494 (0.051)
VPC, MOR	9.1%, 1.73	9.0%, 1.73	11.9%, 1.95	12.1%, 1.95

VPC = Variance Partition Coefficient | MOR = Median Odds Ratio

In fully-adjusted models, frequent use of public transport was found to be consistently associated with better mental health for GHQ caseness (OR 0.85, 95%CI 0.79, 0.91), GHQ-36 (coefficient -0.28, 95%CI -0.41, -0.16), MCS (coefficient 0.45, 95%CI 0.23, 0.66), and WEMWBS (coefficient 0.30, 95%CI 0.19, 0.40). Frequent selection of walking or cycling for journeys of distances less than two to three miles was not associated with GHQ caseness (OR 1.11, 95%CI 0.16, 7.96) and GHQ-36 (coefficient 0.14, 95%CI -0.02, 0.29), but it was associated with lower levels (i.e. less favourable) of MCS (coefficient -0.60, 95%CI -0.85, -0.29), and WEMWBS (coefficient -0.17, 95%CI -0.31, -0.04).

**Fig 2:**
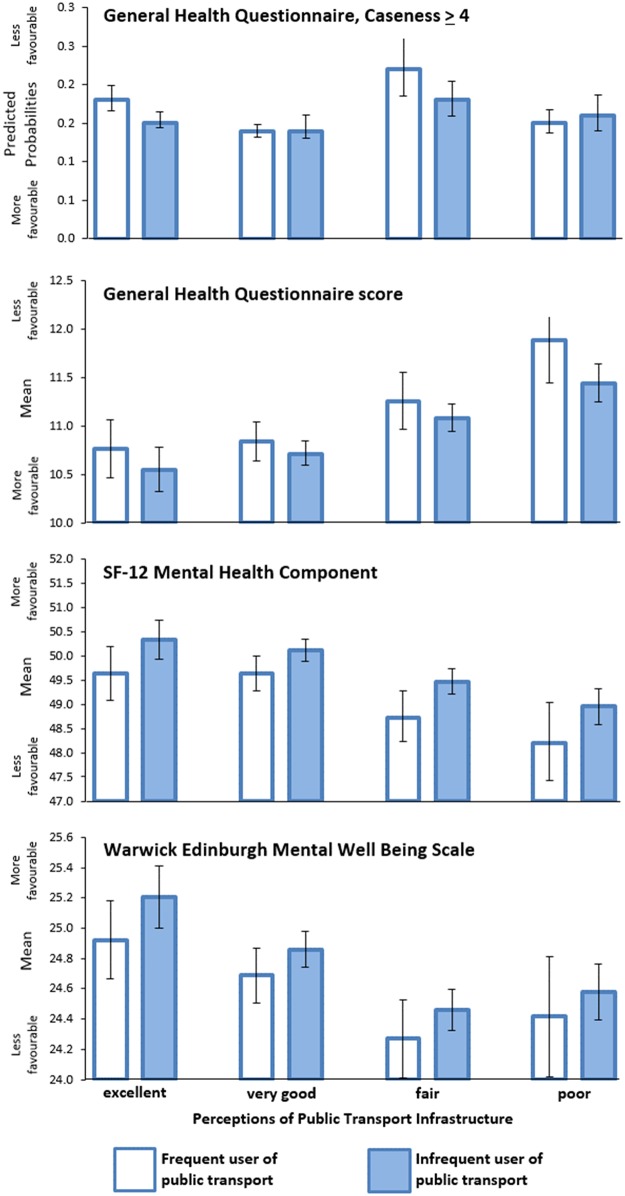
Fully adjusted predictions for the cross-classification of perceptions of public transport and frequency of using of public transport, for 4 contrasting mental health indicators.

The focus in the final set of analyses focussed on the effect modification of observed benefit of public transport use on mental health by PPTI. The fully-adjusted cross-classification of PPTI and use of public transport was associated with poorer mental health in the hypothesised direction (Table 4). Among frequent users of public transport, participants who regarded PPTI as of poor quality were 1.46 times more likely to report poorer mental health according to the GHQ caseness indicator (95%CI 1.11, 1.93), compared to frequent users that regarded PPTI as excellent. This result was replicated across each of the other three indicators of mental health. Substantially weaker associations with mental health indicators were observed for participants expressing poor PPTI who were infrequent users of public transport. Predictions from these fully adjusted models are illustrated in Fig 2. A 5.89 percentage point gap in the GHQ≥4 outcome separated frequent public transport users with poor and excellent PPTI. Similarly, a mean difference of 1.13 gap was observed between the same groups for the GHQ-36, -1.4 for the MCS and -0.5 for the WEMWBS. Poorer mental health was indicative for poorer PPTI also among infrequent users of public transport, though to a demonstrably smaller degree.

**Table 4:** Association between mental health, frequency of public transport use and perceptions of public transport infrastructure.

	GHQ cases ≥ 4	GHQ score (linear)	MCS	WEMWBS
**Fixed Part**	OR (95%CI)	Coefficient (95%CI)	Coefficient (95%CI)	Coefficient (95%CI)
Gender (ref: male)				
female	1.41 (1.32, 1.51)	0.86 (0.75, 0.98)	-1.42 (-1.61, -1.22)	-0.05 (-0.14, 0.05)
Age group (16-19y)				
20-24y	1.04 (0.84, 1.28)	0.80 (0.41, 1.20)	-0.97 (-1.66, -0.29)	-0.38 (-0.71, -0.05)
25-29y	1.06 (0.85, 1.31)	1.04 (0.63, 1.44)	-1.79 (-2.50, -1.08)	-0.56 (-0.91, -0.22)
30-34y	1.06 (0.86, 1.30)	1.12 (0.73, 1.51)	-1.68 (-2.37, -1.00)	-0.30 (-0.63, 0.03)
35-39y	1.01 (0.82, 1.24)	1.15 (0.77, 1.54)	-1.45 (-2.12, -0.77)	-0.21 (-0.54, 0.11)
40-44y	1.00 (0.81, 1.22)	1.26 (0.88, 1.63)	-1.25 (-1.91, -0.59)	-0.11 (-0.43, 0.21)
45-49y	0.98 (0.80, 1.20)	1.30 (0.92, 1.68)	-0.68 (-1.34, -0.02)	-0.10 (-0.42, 0.21)
50-54y	1.01 (0.82, 1.24)	1.23 (0.84, 1.61)	-0.49 (-1.16, 0.17)	0.10 (-0.22, 0.42)
55-59y	0.77 (0.62, 0.95)	0.67 (0.28, 1.06)	0.59 (-0.09, 1.28)	0.67 (0.34, 1.00)
60-64y	0.53 (0.42, 0.67)	-0.06 (-0.47, 0.36)	1.80 (1.07, 2.53)	1.29 (0.94, 1.64)
65-69y	0.43 (0.33, 0.56)	-0.45 (-0.90, 0.00)	2.87 (2.08, 3.65)	1.72 (1.33, 2.10)
70y or older	0.35 (0.27, 0.46)	-0.98 (-1.43, -0.53)	3.01 (2.22, 3.80)	1.74 (1.36, 2.12)
PPTI (ref: excellent PPTI, frequent use of public transport)				
excellent PPTI, infrequent public transport use	0.88 (0.72, 1.07)	-0.21 (-0.58, 0.15)	0.71 (0.06, 1.35)	0.28 (-0.03, 0.59)
very good PPTI, frequent use of public transport	0.92 (0.76, 1.12)	0.08 (-0.28, 0.43)	0.00 (-0.62, 0.62)	-0.24 (-0.54, 0.06)
very good PPTI, infrequent public transport use	0.84 (0.71, 1.00)	-0.05 (-0.37, 0.27)	0.48 (-0.08, 1.04)	-0.06 (-0.33, 0.21)
fair PPTI, frequent use of public transport	1.13 (0.91, 1.41)	0.49 (0.07, 0.90)	-0.90 (-1.63, -0.18)	-0.66 (-1.01, -0.31)
fair PPTI, infrequent public transport use	0.94 (0.79, 1.13)	0.32 (-0.01, 0.65)	-0.17 (-0.75, 0.41)	-0.46 (-0.75, -0.18)
poor PPTI, frequent use of public transport	1.46 (1.11, 1.93)	1.14 (0.59, 1.69)	-1.44 (-2.41, -0.48)	-0.50 (-0.97, -0.04)
poor PPTI, infrequent public transport use	1.15 (0.94, 1.40)	0.67 (0.31, 1.04)	-0.68 (-1.33, -0.04)	-0.34 (-0.66, -0.03)
no opinion PPTI, frequent use of public transport	1.33 (0.62, 2.84)	-0.60 (-2.08, 0.87)	0.08 (-2.50, 2.66)	-0.37 (-1.61, 0.88)
no opinion PPTI, infrequent public transport use	0.84 (0.69, 1.03)	0.10 (-0.26, 0.46)	0.48 (-0.15, 1.11)	-0.02 (-0.33, 0.29)
missing	1.14 (0.93, 1.40)	0.65 (0.27, 1.03)	-1.32 (-1.99, -0.66)	-0.36 (-0.68, -0.04)
Highest educational qualification (ref: Degree)				
Other higher degree	1.03 (0.92, 1.16)	0.17 (-0.04, 0.37)	-0.39 (-0.74, -0.04)	-0.43 (-0.60, -0.26)
A-level	1.04 (0.94, 1.15)	0.18 (0.01, 0.36)	-0.46 (-0.77, -0.16)	-0.56 (-0.71, -0.41)
GCSE	1.06 (0.96, 1.17)	0.24 (0.07, 0.41)	-0.62 (-0.93, -0.32)	-0.93 (-1.08, -0.78)
Other qualification	1.11 (0.98, 1.26)	0.32 (0.10, 0.55)	-0.73 (-1.12, -0.34)	-1.09 (-1.27, -0.90)
No qualification	1.11 (0.98, 1.25)	0.36 (0.14, 0.58)	-1.54 (-1.92, -1.15)	-1.12 (-1.30, -0.93)
Missing	1.03 (0.77, 1.39)	0.36 (-0.17, 0.90)	-1.01 (-1.95, -0.06)	-0.86 (-1.31, -0.40)
Economic status (ref: employed)				
unemployed	1.87 (1.63, 2.15)	1.86 (1.57, 2.15)	-3.36 (-3.86, -2.85)	-1.53 (-1.78, -1.29)
retired	1.27 (1.09, 1.49)	0.35 (0.09, 0.60)	-1.31 (-1.76, -0.86)	-0.08 (-0.30, 0.13)
family care	1.30 (1.14, 1.48)	0.83 (0.57, 1.10)	-2.03 (-2.49, -1.57)	-0.51 (-0.73, -0.28)
student	1.15 (0.95, 1.40)	0.62 (0.25, 0.99)	-0.81 (-1.46, -0.16)	0.01 (-0.30, 0.32)
sick/disabled	2.96 (2.53, 3.47)	4.40 (4.05, 4.76)	-11.32 (-11.94, -10.71)	-3.34 (-3.64, -3.04)
don't know	1.68 (1.09, 2.58)	1.25 (0.38, 2.11)	-2.50 (-4.01, -0.98)	-0.80 (-1.53, -0.07)
Physical functioning (ref: 1 (low))				
2	0.46 (0.41, 0.52)	-2.16 (-2.39, -1.92)	1.27 (0.86, 1.67)	0.86 (0.66, 1.06)
3	0.27 (0.24, 0.30)	-3.50 (-3.74, -3.26)	1.41 (0.99, 1.84)	1.57 (1.36, 1.77)
4	0.13 (0.11, 0.15)	-4.81 (-5.05, -4.56)	3.44 (3.02, 3.87)	2.69 (2.48, 2.90)
5 (high)	0.47 (0.41, 0.53)	-2.06 (-2.31, -1.80)	-4.24 (-4.68, -3.79)	1.36 (1.15, 1.58)
Urban/rural (ref: urban)				
rural	0.86 (0.79, 0.93)	-0.36 (-0.51, -0.22)	1.09 (0.83, 1.34)	0.26 (0.14, 0.39)
**Random Part**				
Regional variance (standard error)	0.005 (0.003)	0.011 (0.009)	0.061 (0.039)	0.020 (0.011)
Variance Partition Coefficient	0.1%	0.04%	0.08%	0.11%
Household variance (standard error)	0.217 (0.046)	3.404 (0.232)	11.131 (0.713)	2.796 (0.167)
Variance Partition Coefficient	6.2%	13.7%	14.6%	15.7%
Person variance (standard error)	not applicable	21.403 (0.269)	64.941 (0.819)	14.967 (0.189)
Variance Partition Coefficient		86.2%	85.3%	84.2%

GHQ = General Health Questionnaire | MCS = SF-12 Mental Health Component | WEMWBS = Warwick Edinburgh Mental Well Being Scale

## Discussion

The physical health implications of active travel are increasingly widely known [7–11]. Over short distances, encouraging walking and cycling rather than driving cars is a policy imperative, but over longer distances, promoting the use of public transport is crucial. It is already recognised that investments in walkability and public transport infrastructure are required to promote more equitable, health promoting built environments. Our study supports this general position, with people who more frequently use public transport tending to have more favourable levels of mental health across four contrasting indicators. But, in line with our hypothesis, PPTI modified this association, wherein frequent users of public transport infrastructure perceived to be lower in quality reported poorer mental health across each indicator. Investments in public transport infrastructure must, therefore, also be perceived by the public as safe, easy to use and fit-for-purpose in order to maximise potential co-benefits for the community.

These findings are novel given the paucity of epidemiological research on active travel via public transport that takes into account PPTI. This is somewhat surprising given prior research from the transport literature that emphasises the relationship between perceptions of quality and subsequent use of public transport. Studies in different parts of the world have shown, for example, that services ought to be designed in ways that accommodate the needs of passengers [37]. That those passengers who have to wait for extended periods for buses or trains prefer to do so in conditions that they feel are comfortable, clean, safe, lit, sheltered from the weather and with service staff present [14]. These attributes as well as a reliable service more generally is likely to be particularly important for people who are frequent users of public transport [13, 38]. As such, it is plausible that for both those who elect to use public transport over a car because it fits their preferences and values and people who have no choice but to use it to get from A to B, perceptions of quality are highly likely to play a potentially powerful role in shaping their wellbeing.

These results also have a bearing for the wider preventive health agenda, since there can be no health without mental health [39]. Provision of public transport infrastructure is a necessary pre-condition to stimulating increases in active travel, but urban planners and other decision makers must take steps to ensure that those investments are attractive to the local populous. Availability does not equate to quality and it is the combination of these elements that are not only crucial to making public transport the default option for longer journeys instead of cars, but also for the protection of mental health for entire populations.

Our study benefits from using large nationally representative data with time-lagged PPTI and confounder variables relative to the mental health and use of public transport indicators, which helped to avoid bias due to reverse causation. The use of four contrasting mental health indicators is also advantageous, providing opportunities to test the consistency of our results across indicators as diverse as cases of minor psychological morbidity to positive mental wellbeing. Multilevel models also demonstrated the relevance of taking into account household clustering in household surveys, which has previously not always been the case.

A possible limitation of our study includes the reliance upon self-reported data, though it is not known whether objective measurement of public transport quality correlates with the inherently subjective PPTI. Previous research comparing objective and subjective measurement of walkability [40] and green space [41] availability suggests the correlation may not be strong. This is an important area for future interdisciplinary research between epidemiologists and transport researchers as it would indicate the extent that interventions should focus upon physical changes in public transport infrastructure and/or marketing strategies to make existing infrastructure more attractive and well-known.
